# Michael D. Waterfield

**DOI:** 10.1042/BCJ20230368

**Published:** 2023-09-21

**Authors:** Julian Downward, Peter J. Parker, Bart Vanhaesebroeck

**Affiliations:** 1 Oncogene Biology Laboratory, The Francis Crick Institute, 1 Midland Road London NW1 1AT, U.K.; 2 Protein Phosphorylation Laboratory, Francis Crick Institute, 1 Midland Road, London NW1 1AT, U.K.; 3 School of Cancer and Pharmaceutical Sciences, New Hunt's House, Guy's Campus, London SE1 1UL, U.K.; 4 UCL Cancer Institute, University College London, 72 Huntley Street, London WC1E 6BT, U.K.


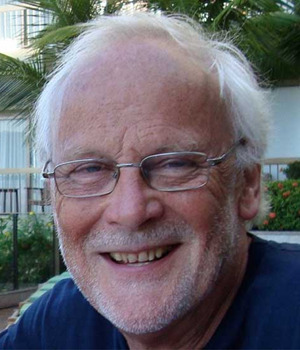


Mike was born on the 14th of May 1941 in Lyndhurst, Hampshire, U.K., in the heart of the New Forest, an area of outstanding natural beauty in southern England. He was one of three children for parents Kathleen and Lesley Waterfield, spending an inquisitive childhood growing up in this idyllic setting. Mike had a formative education at Portsmouth Grammar School, where he developed a passion for chemistry and chemical pathways. After A-levels Mike gained a place at Brunel University in London to study Biochemistry. It was one of the first U.K. universities to offer a year out in industry and this provided him perspective on his academic work, realizing its real-world applications. Following his undergraduate degree at Brunel, Mike completed a PhD at King's College Hospital Medical School in London, working on protein chemistry and enzymology.

In 1967, Mike set out for the United States to do post-doctoral work, first at Harvard, from where he published his first Nature paper on protein sequencing [1] and then to Caltech where he worked with Leroy Hood and Bill Dreyer developing gas-phase protein sequencing. This improved the sensitivity of protein sequencing by a thousand-fold over the best commercially available systems of the time. With this innovation in protein sequencing technology, it became possible for labs to sequence the extensive and important world of low-abundance proteins.

Mike won an American Heart Foundation Fellowship and with some insightful head-hunting from Mike Fried, he was recruited back to the U.K. in 1972 by Michael Stoker to join the Imperial Cancer Research Fund (ICRF) in London, taking his fellowship with him. Bringing his knowledge and skills around advanced protein sequencing techniques to the ICRF was an important initiative in the expanding technological strengths of the institute. The ICRF in return was a very stimulating and exciting place to be — Mike always believed that remarkable opportunities came from having freedom, a critical mass of people and people that inspire you. All this was afforded within the vibrant atmosphere of the ICRF and facilitated by the generous core funding from the institute allowing further developments to be implemented in Mike's expanding laboratory.

Amongst many members of the laboratory recruited in the decade or so that Mike was at the ICRF, the technical duo of Geoff Scrace and Nick Totty played key roles in maintaining, developing and running this state-of-the-art protein sequencing facility. Mike's prescience led him to be one of the first laboratories to implement a protein sequence database, imported from Russ Doolittle, painstakingly curated and kept up-to-date by Geoff and interrogated through Mike's recruitment of the bioinformatician Peter Stockwell. This first-generation state-of-the-art database and the ability to interrogate it were crucial in the breakthroughs that followed.

At ICRF Mike deployed his skills in protein sequencing in a wide range of collaborations; with John Skehel on influenza hemagglutinin [2], with Alan Smith and with Mike Fried on polyoma virus [3], and with a host of other luminaries. Mike then turned his attention at the start of the 1980s to cell growth control and specifically growth factors and their receptors. An initial foray into growth factors came in a collaboration with his ICRF colleague Enrique Rozengurt in an attempt to sequence FDGF [4]; the limited ability to produce this factor from secreted adherent cell cultures proved a step too far even for Mike's hypersensitive instrumentation. However, undeterred, Mike switched to tackling the sequence of PDGF and this proved profound. In-house efforts led from Mike's team by Paul Stroobant, were complemented by approaches to collaborate from the laboratories of Tom Deuel and Calle Heldin, both with track records working with this growth factor. The eventual success of this concerted two-year effort was the generation of the amino-terminal sequence of PDGF. This was a tour-de-force in achievement, but the sequence itself was unremarkable. However, alignment of this sequence with the database held in Mike's lab led Peter Stockwell to find that the sequence coincided almost perfectly with a sequence determined for the retroviral *v-sis* oncogene [5]. The impact of this relationship was recognized immediately — oncogenes were not ‘invented’ by viral evolution to transform cells but rather stolen by viruses on passage through cells. In lay terms, part understood by the press frenzy that followed, cancers could be caused by the hijacking of normal growth control processes and by extrapolation could potentially be targeted therapeutically by intervening in these processes. The dissemination of this groundbreaking work to the wider scientific community was not without its own extraordinary story (see http://blueskiesbenchspace.org/index.php?pag=5 by Kathy Weston) but the impact on cancer research world-wide was wide-ranging.

In Mike's laboratory, amongst all this excitement, was another project that had become the sole domain of a then graduate student, one of the authors here (Julian Downward). Julian was tasked with sequencing not a growth factor but a growth factor receptor, the Epidermal Growth Factor receptor (EGFR). This was again an immense protein purification challenge, albeit greatly facilitated by a monoclonal antibody to the EGFR that Mike had generated in a collaboration with Peter Goodfellow at the ICRF [6]. This project again led to a wider collaborative effort with both Yossi Schlessinger at the Weizmann Institute in Israel who had a shared interest in EGFR, and with Axel Ullrich at Genentech who was one of the most productive cloners in the then-new world of gene cloning (Axel's lab went on to clone the gene for EGFR amongst many others). Julian stuck to his task with extraordinary dedication, despite competition for cold room space with other large-scale protein purifications involving considerable volumes of blood products and cattle brains being employed by others, not to mention the noxious fumes of mercapto-ethanol floating around. Against this backdrop, Julian made a second quite exceptional landmark finding: the EGFR was the origin of the *v-erbB* oncogene encoded by the avian erythroblastosis virus [7]. Here, multiple sequences determined for EGFR lined up with the sequence freshly deposited by Jeff Scrace for this retroviral oncogene. Mike, his team and collaborators had hit the jackpot a second time in the space of months. Linked to the prior finding of the *v-sis*/PDGF relationship, this result unequivocally nailed the relationship between oncogenes and physiological growth controls [8].

The impact of these observations galvanized academia and industry, triggering both an explosion in signal transduction research particularly linked to cancer and spawning a breadth of efforts in targeting druggable transducers, including the EGFR itself and its close relatives such as HER2. Some forty years on we can reflect on how important these groundbreaking findings were to the investment in these efforts and indeed the impact they have had in the clinic. Over fifteen oncology drugs now approved for clinical use target members of the EGF receptor family, including such blockbusters as osimertinib and trastuzumab. For Mike in the short term, these findings triggered a series of efforts relating to the cancer-associated aberrant expression, function, and biochemistry of EGFR involving other members of his laboratory (including: Mark Berger, Charlie Greenfield, Bill Gullick, John Haley, Justin Hsuan, Elaine Mayes and Nigel Whittle) and collaborators (including Yossi and Axel), several of which were published in the pages of this Journal [9,10]. But, Mike's sequencing contributions were far from coming to an end. He continued to bring molecular definition to many prominent signal transducing proteins, typically seeking out the least-redundantly coded tryptic peptide sequences that enabled the cloning of the mRNA/genes and hence complete primary structural definition. It is important to realize that this was long before the Human Genome Organization (HUGO) enabled the look-up identification of gene products. Sequencing projects emanated both from Mike's own laboratory and from collaborations with many laboratories from around the world who beat a path to his door. For example, protein kinase C was first sequenced by one of us (Peter J Parker) in Mike's laboratory [11] and was subsequently cloned by Axel Ullrich and his then colleague Lisa Coussens [12,13].

In 1986 Mike was appointed Director of the Ludwig Institute for Cancer Research at University College London in the Courtauld building adjoining the Middlesex Hospital, recruiting a number of new Group Leaders including one of us (PJP) to this generously funded and well-appointed research facility. Mike continued to pursue EGFR research and likewise the sequencing of signaling proteins. In 1989, Mike turned his attention to the growth factor receptor interacting protein, PI 3-kinase (PI3K). Sarah Morgan in the PJP laboratory at the Ludwig had managed the initial purification of PI3K in work published in this Journal [14,15]. Sequencing followed, and in a collaborative tour-de-force Mike cloned the regulatory subunit and then sequenced and cloned the catalytic subunit [16,17]. These initial cloning efforts were led in Mike's lab by Masayuki Otsu and Ian Hiles, leading to not just the primary sequence definition of this critical signal transducer, but to the establishment of a family of related proteins through additional work by Marketa Zvelebil and a large group of others, including another co-author (Bart Vanhaesebroeck), alongside Alexandre Arcaro, Jan Domin, Ritu Dhand, Mike Fry, Sally Leevers, Lindsay MacDougall, George Panayotou [18,19]. A significant amount of this work was also published in the *Biochemical Journal* [20–22]. This broad family of signal transducers is involved in a wide range of growth and related control processes, and occupied much of Mike's research efforts through the nineties. Mike made a significant commitment in parallel with his discovery science to a drug development programme that he and PJP established in 1995 with the Japanese pharmaceutical company Yamanouchi (now Astellas). This collaborative programme ran for over 5 years, during the course of which Paul Workman and his colleagues at the Division of Cancer Therapeutics joined the U.K. academic team. On reprioritization of resources at Yamanouchi, the oncology assets were returned to the U.K. in 2000 and Mike co-founded the Biotech company Piramed with VC support from JPMorgan Partners and Merlin Biosciences, and very ably led by the CEO Michael Moore. Mike retained strong ties with the company, which was successful in delivering clinical candidates, and the company was bought by Roche which in turn delivered these drugs into the clinic. Several inhibitors against PI3K have now been approved for cancer and rare diseases of overgrowth and immune dysregulation [23].

Towards the end of his research career, Mike stepped down as Institute Director and ran his proteomics laboratory at UCL, making new inroads into cancer proteomics and its application in liquid biopsies. Mike retired in 2008.

Such were Mike's contributions and accomplishments that he received several prestigious awards and honours throughout his career. Mike was a Fellow of the Royal Society, the Royal College of Pathologists, and the Academy of Medical Sciences U.K., and for several years he was one of the most frequently cited scientists in the world. Mike was also awarded the Royal Society's Buchanan Medal in 2002 for his exceptional skill in protein biochemistry which transformed our understanding of cellular pathways in cancer. This has only been awarded to 38 people since its inception in 1897. Notwithstanding these accolades, Mike was most proud of creating an environment where colleagues and students could enjoy their science and enjoy their lives as well. In his Retirement symposium speech at the Royal Society, Mike said ‘I've worked with people who have helped me. I think I've helped people, but actually I've worked with people who have had many ideas and I've just given them the opportunity to pursue them.' At the *Biochemical Journal*, we are proud that many former members of Mike's lab have played key roles in moving the Journal forward, and also to have published a significant amount of the laboratory's excellent work over the years.

Mike passed away on the 11th of May 2023, just three days short of what would have been his 82nd birthday. He leaves an enormous legacy, not just in the landscape of cancer therapies which he did so much to transform, but also in the training and promotion of the careers of so many leading scientists that continue to shape the entire enterprise of cancer research, both in the U.K. and worldwide. His greatest legacy though, is his family, his beloved wife Sal and his daughters, Lucy and Rosie.

## Open Access

Open access for this article was enabled by the participation of The Francis Crick Institute in an all-inclusive *Read & Publish* agreement with Portland Press and the Biochemical Society under a transformative agreement with JISC.
